# Functional Analysis of the Autophagy-Related Gene *OsATG4b* in Rice Grain Chalkiness Regulation

**DOI:** 10.3390/plants14162530

**Published:** 2025-08-14

**Authors:** Zhi Hu, Xiang Han, Yumeng Yuan, Ruishan Xing, Hongchun Liu, Chenming Li, Hongli Shen, Yifan Guo, Shengyuan Sun, Yihao Yang, Min Guo, Changjie Yan

**Affiliations:** 1 Jiangsu Key Laboratory of Crop Genomics and Molecular Breeding/Zhongshan Biological Breeding Laboratory/Key Laboratory of Plant Functional Genomics of the Ministry of Education, College of Agriculture, Yangzhou University, Yangzhou 225009, China; huzhi@yzu.edu.cn (Z.H.); mx120240802@stu.yzu.edu.cn (X.H.); 231701330@stu.yzu.edu.cn (Y.Y.); xingruishan@hotmail.com (R.X.); mz120231341@stu.yzu.edu.cn (H.L.); jy1102ymym@163.com (C.L.); 211802221@stu.yzu.edu.cn (H.S.); mx120190586@stu.yzu.edu.cn (Y.G.); sysun1983@yzu.edu.cn (S.S.); yihao.yang@yzu.edu.cn (Y.Y.); guomin@yzu.edu.cn (M.G.); 2 Co-Innovation Center for Modern Production Technology of Grain Crops of Jiangsu Province, Yangzhou University, Yangzhou 225009, China; 3 Guangling College, Yangzhou University, Yangzhou 225009, China

**Keywords:** rice, grain quality, autophagy, *OsATG4b*

## Abstract

Grain chalkiness is an undesirable trait that significantly compromises rice quality, attracting considerable attention from both consumers and breeders. In this study, we characterized the role of the autophagy-related gene *OsATG4b* in rice grain development. *OsATG4b* was predominantly expressed in the endosperm. Compared with wild-type plants, *OsATG4b*-overexpressing lines exhibited significantly reduced grain chalkiness, whereas *OsATG4b* knockout mutants displayed a marked increase in chalkiness. Importantly, *OsATG4b* had no significant effect on other major agronomic traits. Ultrastructure analysis of the endosperm and evaluation of seed storage components revealed that the chalky endosperm in *OsATG4b* Knockout mutants contained loosely packed starch granules, aberrant protein bodies, and reduced levels of seed storage proteins. Furthermore, gene expression analysis indicated that *OsATG4b* regulates the expression of genes involved in storage protein biosynthesis. Together, these findings demonstrate that *OsATG4b* plays a critical regulatory role in determining grain chalkiness in rice.

## 1. Introduction

Rice (*Oryza sativa* L.) is a staple food crop for more than half of the global population [1]. With ongoing economic development and rising living standards, there is increasing demand for high-quality rice [2]. Rice quality is generally evaluated based on four key attributes: appearance, eating and cooking quality, milling quality, and nutritional value [3,4,5]. Among these, chalkiness refers to the opaque regions in the rice endosperm; it is an undesirable trait for the appearance quality of rice and is also negatively correlated with milling and cooking quality [6,7]. Thus, chalkiness not only reduces consumer preference but also compromises its commercial value.

Chalkiness is a complex quantitative trait controlled by multiple genes and highly sensitive to environmental conditions [8,9]. In particular, elevated temperatures during the grain-filling stage have been shown to significantly increase the proportion of chalky grains [10,11]. Generally, the formation of chalkiness results from loosely packed or abnormally developed starch granules (SGs) and protein bodies (PBs) [12,13]. Notably, starch and storage proteins account for approximately 85% and 10% of the dry weight of rice grains, respectively. Disruptions in the biosynthesis or deposition of these storage substances can lead to structural defects in the endosperm, ultimately manifesting as chalkiness [14,15]. Therefore, understanding the physiological and molecular mechanisms regulating starch and protein accumulation during grain development is essential for improving rice grain quality.

Autophagy is a highly conserved intracellular degradation and recycling process in all eukaryotic organisms, including yeast, plants, and mammals [16,17,18]. In plants, autophagy plays essential roles in a wide range of physiological processes, such as growth, development, immune responses, and senescence. It is also strongly induced under adverse environmental conditions, including nutrient deprivation and various abiotic stresses [18,19,20]. This catabolic process involves the de novo formation of double-membrane vesicles known as autophagosomes, which sequester cytoplasmic components and deliver them to the vacuole for degradation and recycling. The molecular machinery underlying autophagy comprises a set of core autophagy-related (ATG) proteins [21,22]. Among these, the ATG8 and ATG12 conjugation systems are central to autophagosome formation. Specifically, the covalent linkage of ATG8 to the membrane lipid phosphatidylethanolamine (PE) is a critical step required for autophagosome expansion and maturation [23,24,25].

ATG4 is a cysteine protease that plays a dual and indispensable role in the autophagy pathway. It mediates both the initial cleavage of ATG8 to expose a C-terminal glycine residue necessary for lipid conjugation and the subsequent deconjugation of ATG8–PE to allow recycling of ATG8 [26,27,28]. In *Arabidopsis thaliana*, two ATG4 isoforms, AtATG4a and AtATG4b, have been identified. Functional studies have shown that ATG4-mediated autophagy is essential for proper root system development under nutrient-deficient conditions [28]. Moreover, the activity of both AtATG4a and AtATG4b is subject to redox regulation and can be reversibly inhibited under oxidative conditions and hydrogen sulfide [29,30]. In rice, recent studies have shown that the natural variations of an *ATG4* gene, *OsATG4a*, contributes to grain size and weight, playing a role in the divergence of grain size between *indica* and *japonica* subspecies [31]. However, despite these advances, the potential roles of rice *ATG4* genes in the regulation of grain chalkiness remain largely unexplored.

In this study, we aimed to investigate the potential role of the autophagy-related gene *OsATG4b* in regulating rice grain chalkiness. Specifically, we aimed to (1) characterize the spatial and temporal expression pattern of *OsATG4b* during grain development; (2) examine the effects of *OsATG4b* loss-of-function and overexpression on grain chalkiness and endosperm structure; and (3) explore the potential regulatory mechanisms through which *OsATG4b* influences the biosynthesis and accumulation of seed storage substances. These objectives will provide new insights into the physiological roles of autophagy in rice grain quality formation.

## 2. Results

### 2.1. Identification of Rice OsATG4b Protein

**Figure 1:**
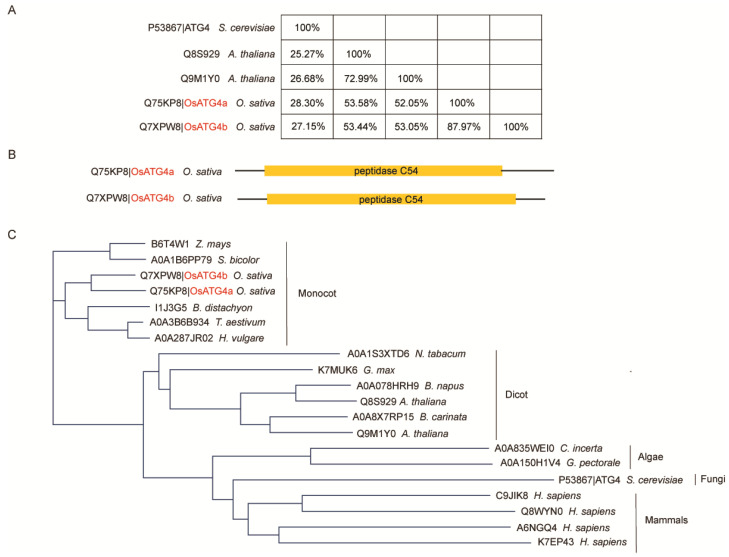
Comparative analysis of ATG4 proteins across various species. (**A**) Protein identity matrix of ATG4 homologs from *Oryza sativa* (*O. sativa*), *Arabidopsis thaliana* (*A. thaliana*), and *Saccharomyces cerevisiae* (*S. cerevisiae*). Pairwise sequence identity was calculated based on amino acid alignment. (**B**) Domain architecture of *O. sativa* ATG4 homologs. Schematic representation of the two rice ATG4 proteins, *OsATG4a* (Q75KP8) and *OsATG4b* (Q7XPW8), showing the conserved Peptidase C54 domain (yellow). (**C**) Phylogenetic tree of ATG4 proteins based on amino acid sequences. The tree reveals the evolutionary relationships among ATG4 homologs from different taxa, showing clear clades for monocots, dicots, and non-plant species (algae, fungi, and mammals). The *OsATG4a* and *OsATG4b* proteins are highlighted in red.

To identify ATG4 homologs in rice, BLASTP searches were conducted against the UniProt database using the yeast ATG4 protein sequence as a query. Two rice homologs, *OsATG4a* and OsATG4b, were identified, exhibiting 28.3% and 27.15% sequence identity with yeast ATG4, respectively (Figure 1A). These two rice proteins share 87.97% sequence similarity with each other and possess a predicted Peptidase C54 catalytic domain, a conserved feature of the ATG4 protein family (Figure 1A,B). To further investigate the evolutionary conservation of ATG4 proteins, additional homologs were retrieved from representative species of algae, plants, and mammals. We performed a phylogenetic analysis based on their amino acid sequences (Figure 1C). The broad distribution of ATG4 homologs among plants, fungi, algae, and animals underscores the conserved role of ATG4 in autophagy throughout eukaryotic evolution.

### 2.2. Expression Patterns of OsATG4a and OsATG4b

**Figure 2:**
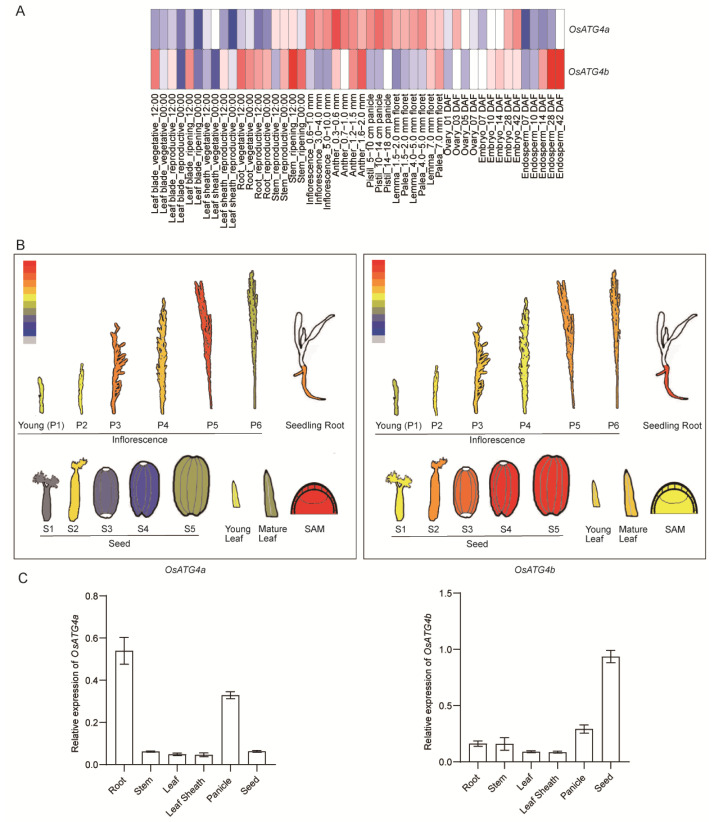
Expression profiles of *OsATG4a* and *OsATG4b* in various tissues and developmental stages of rice. (**A**) Heatmap showing the expression levels of *OsATG4a* and *OsATG4b* across multiple rice tissues at different developmental stages. Data were retrieved from RiceXPro accessed on 2 March 2024 (https://ricexpro.dna.affrc.go.jp). (**B**) Tissue-specific expression patterns of *OsATG4a* and *OsATG4b*, visualized using the ePlant platform accessed on 2 March 2024 (http://bar.utoronto.ca/eplant_rice/). (**C**) qRT-PCR analysis of *OsATG4a* and *OsATG4b* expression in different rice tissues, including root, stem, leaf sheath, leaf, panicle, and seed. Expression levels are normalized to the rice *ACTIN1* gene. Data represent means ± SD (*n* = 3). In A and B, red color indicates relatively high expression levels.

To explore the expression patterns of *OsATG4a* and *OsATG4b*, we first examined public transcriptome datasets. Tissue-specific expression profiles retrieved from the RiceXPro database [32] showed distinct patterns for the two genes across various rice tissues and developmental stages (Figure 2A). Specifically, *OsATG4a* was predominantly expressed in reproductive organs such as the inflorescence, pistil, and lemma, whereas *OsATG4b* showed higher expression levels in the root, stem, and late-stage endosperm. Similar trends were observed using data from the eFP Rice database (Figure 2B).

To obtain direct experimental evidence, we next performed quantitative real-time PCR (qRT-PCR) analysis using RNA extracted from different tissues of field-grown wild-type rice, including root, stem, leaf sheath, panicle, and seed. As shown in Figure 2C, both *OsATG4a* and *OsATG4b* were expressed in all examined tissues. Notably, qRT-PCR results revealed that *OsATG4a* was highly expressed in the panicle, while *OsATG4b* showed markedly higher expression in seeds. These results not only confirm the transcriptome-based observations but also provide direct evidence that the two genes exhibit distinct tissue-specific expression patterns, suggesting potentially divergent functional roles during rice development.

### 2.3. Generation and Phenotypic Characterization of OsATG4a and OsATG4b Knockout Mutants

**Figure 3:**
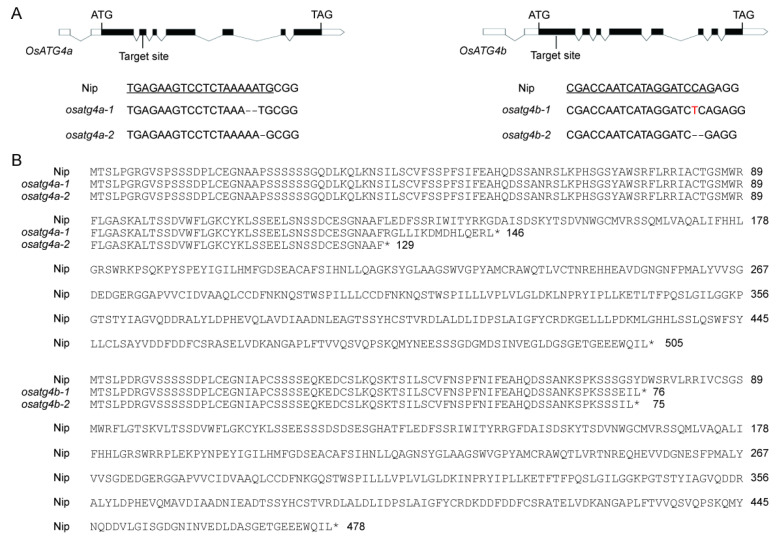
Generation of *OsATG4a* and *OsATG4b* knockout mutants. (**A**) Schematic representation of CRISPR/Cas9 target sites in *OsATG4a* and *OsATG4b* genes. Black boxes represent exons, and arrows mark the positions of target sites relative to the start codon (ATG) and stop codon (TAG). Wild-type (Nip) sequences and corresponding mutant alleles (*osatg4a-1*, *osatg4a-2*, *osatg4b-1*, and *osatg4b-2*) are shown. Dashes indicate deleted nucleotides. Target sequences are underlined; inserted base is displayed in red. (**B**) Predicted amino acid sequences of *OsATG4a* and *OsATG4b* proteins in wild-type and mutant lines. Frameshift mutations in mutants result in premature stop codons, leading to truncated proteins. Asterisks indicate stop codons. Numbers on the right correspond to the amino acid positions.

To investigate whether *OsATG4a* and *OsATG4b* have distinct functions in rice, we generated knockout mutants in the Nipponbare (Nip) background using CRISPR/Cas9-mediated genome editing. Target sites were designed within the coding regions of both genes (Figure 3A). Two *OsATG4a* mutant alleles, *osatg4a-1* and *osatg4a-2*, harbored 2 bp and 1 bp deletions (Figure 3A), respectively, leading to premature stop codons at the 146th and 129th amino acid positions (Figure 3B). Similarly, two *OsATG4b* mutant alleles, *osatg4b-1* and *osatg4b-2*, carried a 1 bp insertion and a 2 bp deletion (Figure 3A), respectively, resulting in premature stop codons at the 75th and 89th amino acid positions (Figure 3B).

**Figure 4:**
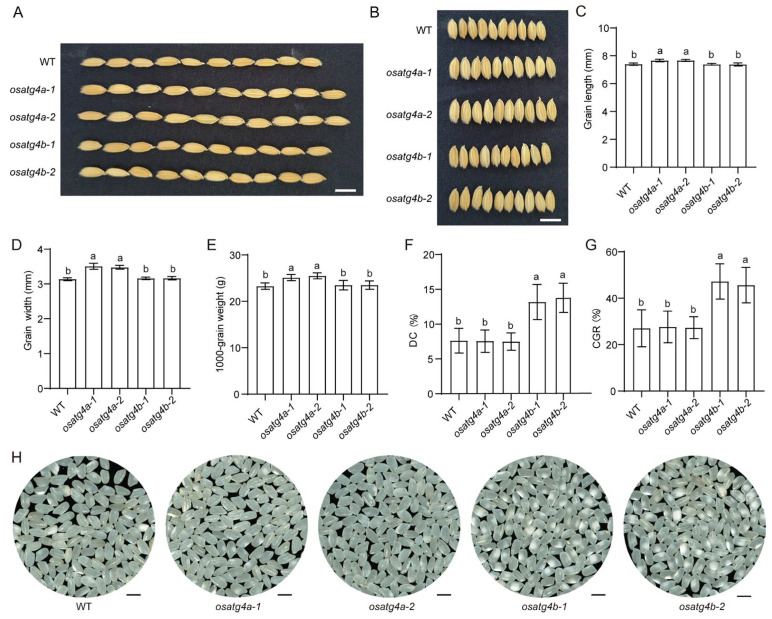
Grain morphology and chalkiness in *OsATG4a* and *OsATG4b* knockout mutants. (**A**) Comparison of grain length among WT, *osatg4a* and *osatg4b* plants. Scale bars, 0.8 cm. (**B**) Comparison of grain width among WT, *osatg4a* and *osatg4b* plants. Scale bars, 0.8 cm. (**C**) Comparative analysis of grain length at maturity in (**A**). Values are means ± SD (*n* = 50). (**D**) Comparative analysis of grain width at maturity in (**B**). Values are means ± SD (*n* = 50). (**E**) Evaluation of 1000-grain weight among WT, *osatg4a* and *osatg4b* plants. (**F**,**G**) Assessment of grain chalkiness traits, including degree of chalkiness (DC) and chalky grain rate (CGR) among WT, *osatg4a* and *osatg4b* plants. (**H**) Morphological appearance of polished grains among WT, *osatg4a* and *osatg4b* plants. Scale bar = 5 mm. Data are presented as means ± SD (*n* = 10). Different letters indicate significant differences (*p* < 0.05), as determined by one-way ANOVA with Tukey’s multiple comparison test.

Compared with the wild type, *OsATG4a* knockout mutants exhibited significantly increased grain length, grain width, and 1000-grain weight (Figure 4A–E), consistent with previous findings identifying *OsATG4a* as a negative regulator of grain size and weight in rice [31]. In contrast, the knockout of *OsATG4b* did not result in significant changes in these grain morphology traits (Figure 4A–E). Given the high expression of *OsATG4b* in the endosperm, we further examined grain appearance in the *OsATG4b* knockout mutants. The *osatg4b-1* and *osatg4b-2* mutants displayed markedly increased chalkiness compared to the wild type, as reflected by higher chalky grain rate (CGR) and degree of chalkiness (DC) (Figure 4F–H). Conversely, *OsATG4a* knockout mutants showed no significant difference in chalkiness (Figure 4F–H).

**Figure 5:**
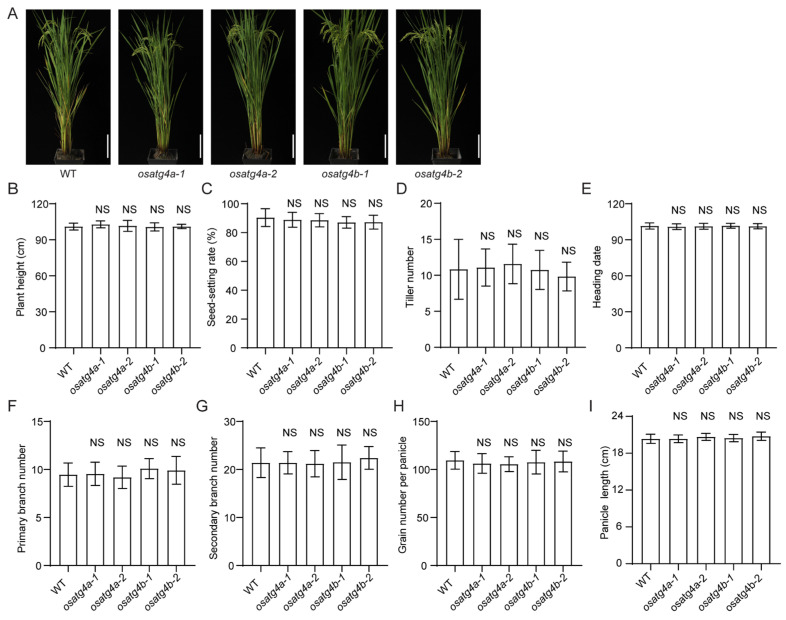
Agronomic trait analysis in *OsATG4a* and *OsATG4b* knockout mutants. (**A**) Photographs of wild-type (WT) plants, *osatg4a* mutants, and *osatg4b* mutants grown in natural conditions. Scale bars, 25 cm. (**B**–**I**) Comparison of plant height, seed-setting rate, tiller number, heading date, primary branch number, secondary branch number, grain number per panicle, and panicle length among WT, *osatg4a*, and *osatg4b* plants. Data are presented as means ± SD (*n* = 10). Significant difference was determined by Student’s *t*-test. NS, not significant.

Furthermore, no significant differences were observed in major agronomic traits—including plant height, seed-setting rate, tiller number, heading date, number of primary and secondary branches, panicle length, and grain number per panicle—between the *OsATG4a* and *OsATG4b* knockout mutants and wild-type plants (Figure 5).

### 2.4. Overexpression of OsATG4b Decreases Grain Chalkiness

**Figure 6:**
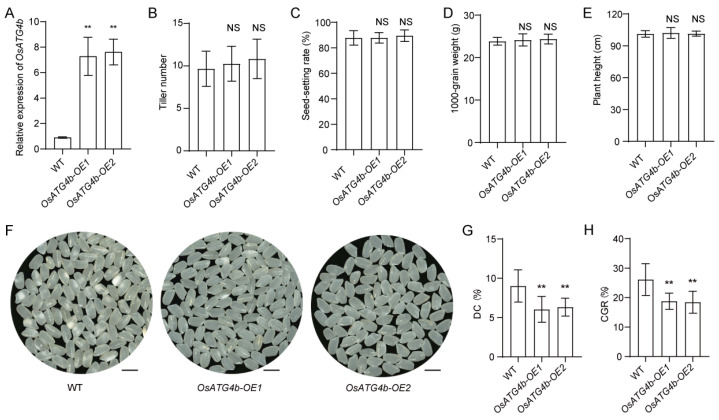
Phenotypic characterization of *OsATG4b* overexpression lines. (**A**) Relative expression levels of *OsATG4b* in transgenic overexpression (OE) lines as determined by qRT-PCR. (**B**–**E**) Agronomic trait comparisons between WT and *OsATG4b*-*OE* lines, including tiller number, seed-setting rate, 1000-grain weight, and plant height. (**F**) Grain appearance from WT and *OsATG4b-OE* lines. (**G**,**H**) Chalkiness evaluation of WT and OE lines, including DC and CGR. Data are means ± SD (*n* = 10). Statistical significance was determined using Student’s *t*-test: *p* < 0.01 (**); NS, not significant.

To further investigate the role of *OsATG4b* in regulating grain chalkiness, transgenic lines overexpressing *OsATG4b* under the control of the ubiquitin promoter were generated in the Nip background. Quantitative RT-PCR analysis confirmed that *OsATG4b* transcript levels were significantly elevated in the overexpression (OE) lines, with OE1 and OE2 showing 8.0- and 8.4-fold increases, respectively, compared with the wild type (Figure 6A). Under field conditions, the OE lines did not show significant differences from the wild-type plants in key agronomic traits, including tiller number, 1000-grain weight, seed-setting rate, and plant height (Figure 6B–E). However, the OE lines produced more transparent grains and exhibited significantly reduced CGR and DC relative to the wild type (Figure 6F–H).

### 2.5. Altered Structure and Composition of Seed Storage Substances in OsATG4b Endosperm

**Figure 7:**
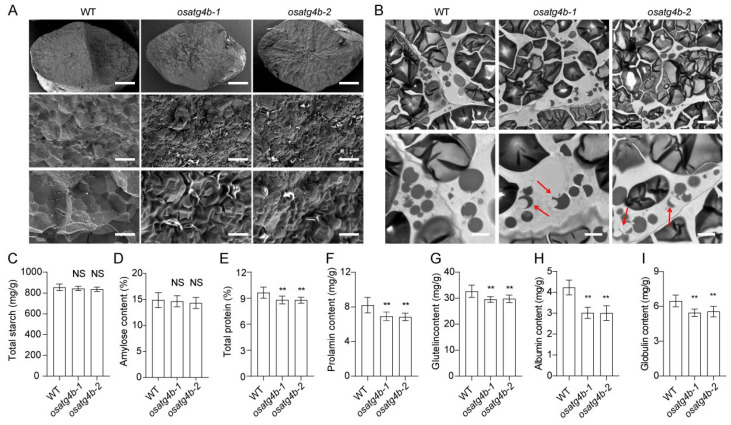
*OsATG4b* affects starch granule and protein body morphology, as well as seed storage protein accumulation. (**A**) Scanning electron microscopy images of transverse sections from mature seeds of WT and *osatg4b* mutants, showing altered endosperm structure. Scale bars: 500 µm (top), 10 µm (middle), 5 µm (bottom). (**B**) Transmission electron microscopy analysis of developing endosperm cells at 13 days after flowering (DAF). Scale bars: 5 µm (top), 2 µm (bottom). Red arrows indicate abnormal protein bodies. (**C**,**D**) Quantification of total starch content and apparent amylose content in mature seeds. (**E**–**I**) Comparison of total protein, albumin, glutelin, prolamin, and globulin contents among WT and *osatg4b* seeds. Data are presented as means ± SD (*n* = 10). Statistical significance was determined using Student’s *t*-test: *p* < 0.01 (**); NS, not significant.

Grain chalkiness is often associated with alterations in endosperm ultrastructure and the composition of seed storage components. To evaluate these changes, scanning electron microscopy was used to examine the morphology of SGs in the endosperm of wild-type and *OsATG4b* mutant plants. In wild-type grains, SGs were regularly polyhedral and tightly packed. In contrast, SGs in the *OsATG4b* knockout mutants were smaller, round, and loosely arranged (Figure 7A). To further assess the structure of PBs, transmission electron microscopy was conducted on endosperm sections at 12 days after flowering (DAF). Wild-type endosperm displayed abundant and well-defined PBs, including irregularly shaped PBII and spherical PBI structures (Figure 7B). However, aberrant PBs were observed in the endosperm of *OsATG4b* mutants (Figure 7B). These results indicate that the morphology of both SGs and PBs was disrupted in the absence of *OsATG4b* function.

To examine whether these structural abnormalities were associated with altered storage compound accumulation, we quantified the storage component content of mature seeds. While total starch and amylose contents were comparable between *OsATG4b* mutants and Nip, total protein content was significantly reduced in the mutants (Figure 7C–E). Further analysis of individual protein fractions showed significant reductions in glutenin, prolamin, albumin, and globulin levels (Figure 7F–I). These findings suggest that the increased grain chalkiness observed in *OsATG4b* mutants is associated with defective accumulation and organization of storage substances in the endosperm.

### 2.6. OsATG4b Mutation Alters Expression of Storage Protein Genes Without Affecting Starch Biosynthesis

**Figure 8:**
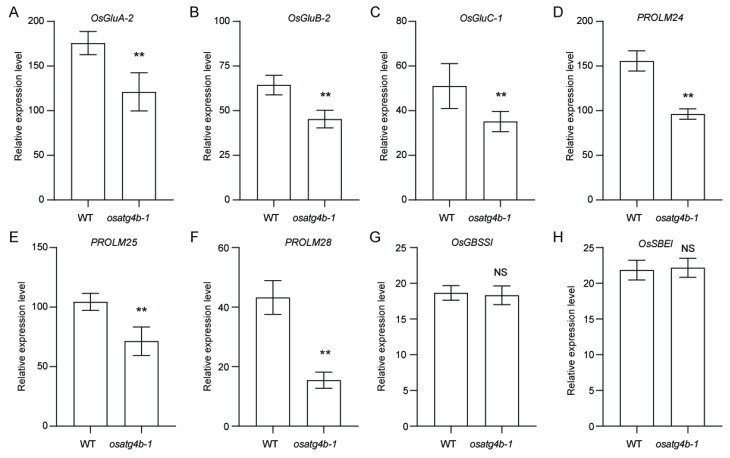
qRT-PCR analysis of genes involved in seed storage protein and starch metabolism. (**A**–**H**) Relative expression levels of *OsGluA-2*, *OsGluB-2*, *OsGluC-1*, *PROLM24*, *PROLM25*, *PROLM28*, *OsGBSSI*, and *OsSBEI* in WT and *osatg4b-1*. Data represent means ± SD (*n* = 3). Statistical significance was determined using Student’s *t*-test: *p* < 0.01 (**); NS, not significant.

To investigate the basis of altered grain chalkiness phenotype in the *osatg4b* mutant, qRT-PCR analysis was performed on selected genes involved in storage protein and starch biosynthesis. The expression levels of *OsGluA-2*, *OsGluB-2*, *OsGluC-1*, *PROLM24*, *PROLM25*, and *PROLM28* were significantly downregulated in *osatg4b-1* relative to WT (Figure 8A–F). In contrast, *OsGBSSI* and *OsSBEI* transcript levels remained unchanged (Figure 8G,H), suggesting that *OsATG4b* primarily regulates storage protein-related gene expression during seed development.

## 3. Discussion

ATG4 plays a central role in the autophagy pathway by processing ATG8 precursors, a critical step required for autophagosome formation and recycling [26,28,29]. To better understand the functional significance and potential divergence of ATG4 homologs in rice, we investigated the roles of *OsATG4a* and OsATG4b. Despite their high amino acid sequence similarity, *OsATG4a* and *OsATG4b* have distinct expression patterns. *OsATG4a* is preferentially active in reproductive tissues, whereas *OsATG4b* is predominantly expressed in the endosperms (Figure 2). This spatial divergence in expression suggests that the two genes may have evolved specialized roles in rice grain development. Functionally, *OsATG4a* regulates grain size, while *OsATG4b* is involved in grain chalkiness (Figure 4 and Figure 5). The strong endosperm-specific expression of *OsATG4b* coincides with the timing of storage reserve accumulation, a critical period when protein and starch homeostasis directly influences endosperm texture. The disruption of *OsATG4b* led to altered protein body morphology, which contributes to increased chalkiness. In contrast, *OsATG4a*, with little to no endosperm expression, does not affect chalkiness, further supporting the tissue-specific functional divergence of the two homologs. Together, these findings highlight that *OsATG4b*’s endosperm-enriched expression underpins its specific role in modulating grain chalkiness, distinguishing it mechanistically and functionally from *OsATG4a*.

Autophagy is known to play essential roles in plant growth, development, and stress responses [33]. In rice, autophagy has been implicated in diverse physiological processes such as nutrient remobilization, leaf senescence, pollen maturation, flowering, and grain size [31,34,35,36,37,38]. Despite these advances, the regulatory mechanisms governing autophagy activity in specific tissues, such as the endosperm, remain poorly understood. In particular, the grain quality regulation of autophagy during grain development is still largely unexplored. Here, we found that loss of function of *OsATG4b* disrupted the morphology of starch granules and the organization of protein bodies in the endosperm, accompanied by a marked reduction in total protein content (Figure 7). Notably, the expression levels of multiple storage protein genes were significantly downregulated in *osatg4b* mutants (Figure 8), indicating that *OsATG4b* may contribute to grain chalkiness by modulating the expression or accumulation of storage proteins during grain filling. Interestingly, although total starch content appeared unaffected, the disruption of protein body structure and abnormal accumulation of loosely packed starch granules likely altered the light-scattering properties of the endosperm, resulting in a chalky appearance. This suggests that proper protein body organization is essential for maintaining the dense, translucent endosperm structure. Given the known roles of autophagy in endoplasmic reticulum (ER) quality control and storage protein turnover [39,40,41], it is plausible that *OsATG4b* regulates grain chalkiness through autophagy-mediated regulation of protein homeostasis in the endosperm. To directly confirm the involvement of autophagic processes, future studies could employ GFP-ATG8 marker lines to monitor autophagosome formation and flux in the developing endosperm, or assess the accumulation of selective autophagy substrates. In addition, analysis of ER stress markers and ultrastructural examination using transmission electron microscopy could further elucidate the role of autophagy in maintaining ER function and protein body integrity during grain filling.

Chalkiness is a complex, quantitative trait regulated by multiple genetic loci and influenced by environmental conditions. Numerous QTLs associated with grain chalkiness have been identified through biparental populations and genome-wide association studies (GWAS), across all 12 rice chromosomes [5]. For example, *Chalk5* was shown to influence grain chalkiness by regulating pH homeostasis in developing seeds [42], while natural variation in *WCR1* affects redox balance in the endosperm and alters chalkiness phenotypes [43]. Other studies identified *WBR7* and *Chalk9* as key regulators of grain chalkiness through their effects on the accumulation and organization of seed storage components [44,45]. In this study, we identified *OsATG4b* as a novel gene involved in the regulation of endosperm chalkiness through a reverse genetics approach. Unlike previously reported chalkiness-associated genes, *OsATG4b* is functionally related to autophagy, a cellular degradation and recycling process that has not been directly linked to grain chalkiness before. Our phenotypic and cytological analyses revealed that loss of *OsATG4b* function disrupts the organization of protein bodies and starch granules, leading to a chalky endosperm phenotype.

Considering its specific expression in endosperm and its functional impact on chalkiness, *OsATG4b* holds promise as a genetic target for improving rice grain quality. Overexpression of *OsATG4b* significantly reduced grain chalkiness without negatively affecting key agronomic traits (Figure 6), highlighting its potential utility in molecular breeding programs aimed at enhancing rice appearance quality and commercial value. Our findings reveal a previously uncharacterized role for *OsATG4b* in endosperm development, likely through selective modulation of storage protein pathways, and broaden the known functional repertoire of autophagy-related genes in crop species.

Despite these insights, several key questions remain. It is yet to be determined whether *OsATG4b* directly regulates the transcription of storage protein genes or acts indirectly through autophagy-mediated ER quality control. Given the known role of autophagy in maintaining ER homeostasis, and our observation of altered protein body morphology and increased chalkiness in *osatg4b* mutants, it is plausible that *OsATG4b* affects storage protein accumulation indirectly by modulating ER stress responses and protein turnover. In particular, disrupted autophagic flux may lead to the accumulation of misfolded proteins in the ER, triggering unfolded protein responses that, in turn, alter the expression or translation efficiency of storage protein genes. Additionally, potential crosstalk between *OsATG4b* and transcriptional regulators of endosperm development, such as *RISBZ1* and *RPBF* [46,47,48], remains to be elucidated. Whether *OsATG4b* affects these transcription factors through post-translational modifications, trafficking, or autophagy-dependent degradation is an intriguing possibility. Future studies employing protein–protein interaction assays, real-time tracking of autophagosome dynamics, and analyses of ER stress markers will be critical for dissecting the precise molecular mechanisms by which *OsATG4b* influences storage protein accumulation and grain quality in rice.

## 4. Materials and Methods

### 4.1. Plant Materials and Growth Conditions

The rice cultivar used in this study was the *japonica* variety Nipponbare (Nip). Wild-type and transgenic lines were cultivated under natural field conditions either at the experimental paddy fields of Yangzhou University (Yangzhou, China) or in Lingshui, Hainan Province, depending on seasonal schedules. All plants were cultivated during the normal rice-growing seasons. For each genotype, plants were arranged in two rows with 10 plants per row. The spacing between plants within each row was 18 cm, and the distance between adjacent rows was 25 cm. Field management followed standard agricultural practices. All lines were grown under similar conditions to ensure comparability in phenotypic evaluation.

### 4.2. Identification of ATG4 Homologs and Phylogenetic Analysis

To identify ATG4 homologs across eukaryotic species, the amino acid sequence of *Saccharomyces cerevisiae* ATG4 was retrieved from the NCBI protein database and used as a query for BLASTP searches against the UniProtKB database (https://www.uniprot.org/). An E-value cutoff of 1 × 10^−5^ was applied to select candidate sequences with significant homology. Homologous sequences showing high similarity were retained for further analysis. Multiple sequence alignments of the retrieved proteins were performed using the UniProt Align tool (Clustal X 2.1) to ensure accurate comparison of conserved regions.

For phylogenetic analysis, amino acid sequences were aligned using ClustalW2 [49] with default parameters, followed by manual refinement of misaligned regions. A phylogenetic tree was then constructed using the maximum likelihood (ML) method implemented in MEGA11 (version 11.0.13) software [50]. The LG+G+I substitution model was used based on model testing, and a total of 1000 rapid bootstrap replicates were performed to assess branch support and tree topology reliability.

### 4.3. CRISPR/Cas9-Mediated Knockout of OsATG4a and OsATG4b

To generate knockout mutants of *OsATG4a* and *OsATG4b*, a CRISPR/Cas9 system based on the binary vector pYLCRISPR/Cas9-MH was employed. Gene-specific 20 bp guide RNA (gRNA) target sequences were designed using the CRISPRdirect online tool to ensure high specificity [51]. Synthesized oligonucleotides corresponding to the gRNA sequences were annealed, digested, and ligated into an intermediate CRISPR/gRNA vector. The resulting gRNA expression cassettes were then cloned into the final pYLCRISPR/Cas9-MH vector. The recombinant plasmids were introduced into *Agrobacterium tumefaciens* strain EHA105 via electroporation. Transformed colonies were then used for *Agrobacterium*-mediated transformation of Nip calli. To confirm the presence of the transgene, genomic DNA was extracted from putative transgenic and wild-type plants, and PCR was performed using specific primers targeting the hygromycin resistance gene. PCR products were separated on a 1% agarose gel and visualized with ethidium bromide staining. Representative results are shown in Figure S1A,B. To verify gene editing events, genomic DNA was extracted from T0 transgenic plants, and the regions flanking the target sites were amplified by PCR using gene-specific primers. PCR amplicons were subjected to Sanger sequencing, and resulting chromatograms were analyzed using the online tool DSDecode [52] to identify insertions or deletions (indels) at the target sites. Primers sequences used for genotyping are listed in Table S1.

### 4.4. Construction of OsATG4b Overexpression Lines

For the generation of *OsATG4b* overexpression lines, the full-length coding sequence of *OsATG4b* was amplified from Nip cDNA and cloned into the pCAMBIA1301 vector under the control of the maize ubiquitin promoter. The resulting overexpression construct was introduced into *Agrobacterium tumefaciens* strain EHA105 and subsequently used for *Agrobacterium*-mediated transformation of Nip calli. To confirm transgene integration, PCR detection of the hygromycin resistance gene was performed, and representative results are shown in Figure S1C. Independent T_1_ transgenic lines were obtained and analyzed for expression levels of *OsATG4b* by qRT-PCR. Lines exhibiting high *OsATG4b* transcript levels were selected for further phenotypic characterization. Primer sequences used for cloning and expression analysis are listed in Table S1.

### 4.5. Expression Analysis by Quantitative RT-PCR

To analyze the expression patterns of *OsATG4a* and *OsATG4b* in various rice tissues, total RNA was extracted from roots, stems, leaves, leaf sheaths, panicles, and developing seeds of Nip using the RNA Easy Fast Plant Tissue Kit (DP452, TIANGEN Biotech, Beijing, China) according to the manufacturer’s protocol. For each sample, 0.5 μg of total RNA was reverse-transcribed into first-strand cDNA using the FastKing One Step RT-PCR Kit (KR123, TIANGEN Biotech, Beijing, China). qRT-PCR was conducted using the CFX96 Real-Time PCR Detection System (Bio-Rad, Hercules, CA, USA) and RealUniversal Color PreMix (SYBR Green) (FP201, TIANGEN Biotech, Beijing, China). One-fifth of the synthesized cDNA was used as the template for each reaction. *OsACTIN1* (*LOC_Os03g50885*) was used as an internal reference gene for normalization. Primer sequences used in the analysis are listed in Table S1.

### 4.6. Measurement of Phenotype Data

Agronomic traits were evaluated at maturity in field-grown rice plants. Traits including plant height, tiller number, and heading date were recorded at the flowering stage, while primary and secondary branch number, grain number per panicle, seed-setting rate, grain length, grain width, and 1000-grain weight were measured after physiological maturity. Grain length and width were determined using a digital caliper. For 1000-grain weight, approximately 500 fully filled grains per plant were counted and weighed, and the value was calculated proportionally. CGR and DC were determined using a ScanMaker grain appearance analyzer (Microtek, Zhongjing Technology, Shanghai, China) based on random samples of over more than 100 dehulled grains per plant.

For the analysis of seed storage substances, mature grains were dehulled and ground into fine flour using a laboratory mill. Total starch and amylose contents were determined according to the method described by Yang et al. [53]. Total protein content was measured using the Kjeldahl nitrogen determination method, and protein concentrations were calculated using the appropriate nitrogen-to-protein conversion factor. Endosperm storage proteins—including albumin, globulin, prolamin, and glutelin—were extracted and quantified as previously described [4]. All measurements were performed using three independent biological replicates.

### 4.7. Transmission Electron Microscopy

To investigate ultrastructural changes in the developing endosperm, transmission electron microscopy was performed on wild-type and *osatg4b* mutant seeds collected at 13 DAF. Sample preparation was conducted following the protocol described previously [38], with slight modifications. Briefly, developing seeds were harvested and immediately fixed overnight at 4 °C in a solution containing 4% (*v*/*v*) paraformaldehyde and 0.1% (*v*/*v*) glutaraldehyde. Following fixation, samples were dehydrated through a graded ethanol series (30%, 50%, 70%, 80%, 90%, and 100%) and embedded in resin. Ultrathin sections were prepared using an ultramicrotome and post-stained with 2% uranyl acetate. Sections were then examined using a transmission electron microscope equipped with a 200 kV LaB_6_ electron source and an FEI Eagle 4k CCD camera. At least three biological replicates were analyzed to ensure reproducibility of the observations.

### 4.8. Scanning Electron Microscopy

To examine the morphology of starch granules in mature rice endosperm, scanning electron microscopy was performed on wild-type and *osatg4b* mutant grains. Sample preparation and imaging were carried out according to a previously described protocol [43], with minor modifications. Mature, dehulled rice grains were transversely fractured at the central region of the endosperm using a sterile razor blade. The fractured samples were mounted on aluminum stubs and sputter-coated with a thin layer of gold under vacuum conditions to enhance conductivity. Imaging was conducted using a Carl Zeiss EVO SEM 300 system (Carl Zeiss AG, Oberkochen, Germany) at an accelerating voltage of 10 kV and a spot size of 30 nm. The surface structures of starch granules were observed and recorded. For each genotype, at least three independent biological replicates were analyzed.

### 4.9. Statistical Analysis

All experiments were independently replicated at least three times. Statistical analyses were performed using two-tailed Student’s *t*-test via GraphPad Prism version 8 (GraphPad Software Inc., San Diego, CA, USA). Statistical significance was defined as *p* < 0.01 (**) and *p* > 0.05 (NS: no significant difference); when comparing multiple groups, statistical significance was determined by one-way analysis of variance (ANOVA) with Tukey’s multiple comparisons test.

## 5. Conclusions

We identified *OsATG4b* as a key autophagy-related gene regulating grain chalkiness in rice. It is predominantly expressed in the developing endosperm. Knockout of *OsATG4b* increased chalkiness and disrupted the organization of starch granules and protein bodies, while overexpression reduced chalkiness and enhanced storage protein accumulation. Furthermore, *OsATG4b* modulates the expression of genes involved in protein biosynthesis during seed development. These findings demonstrate that *OsATG4b* plays a crucial role in endosperm development and grain appearance quality, providing new insights into the contribution of autophagy to rice grain quality and offering a potential target for genetic improvement.

## Data Availability

The data used in this study are available within the article and its accompanying Supplementary Materials.

## Supplementary Materials

The following supporting information can be downloaded at: https://www.mdpi.com/article/10.3390/plants14162530/s1, Table S1: Primers used in this study. Figure S1: PCR verification of transgenic rice lines by amplification of the hygromycin resistance gene. (**A**) Detection of *OsATG4a* knockout lines. (**B**) Detection of *OsATG4b* knockout lines. (**C**) Detection of *OsATG4b* overexpression lines. ‘+’ indicates the positive control, ‘−’ indicates the wild-type negative control, and ‘O’ represents the no-template control. M: DNA marker; numbers indicate independent transgenic lines.
