# Supplementary material for: Functional Analysis of the Autophagy-Related Gene OsATG4b in Rice Grain Chalkiness Regulation
Source: Plants (Basel). 2025 Aug 14;14(16):2530. doi: 10.3390/plants14162530 (PMC12389691; doi:10.3390/plants14162530)
Supplement: Supplementary file 1 [file plants-14-02530-s001.zip › Supplementary Figure.pdf]

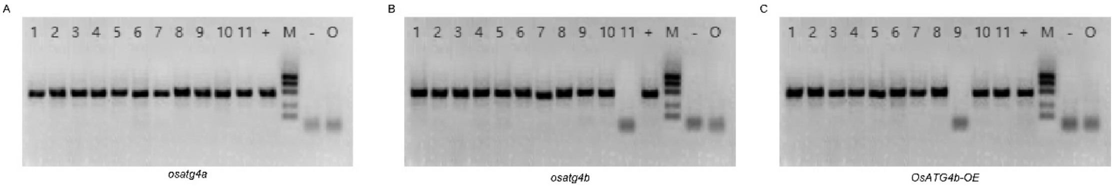

Figure S1: PCR verification of transgenic rice lines by amplification of the hygromycin resistance gene. (**A**) Detection of *OsATG4a* knockout lines. (**B**) Detection of *OsATG4b* knockout lines. (**C**) Detection of *OsATG4b* overexpression lines. '+' indicates the positive control, '-' indicates the wild-type negative control, and 'O' represents the no-template control. M: DNA marker; numbers indicate independent transgenic lines.
